# R306465 is a novel potent inhibitor of class I histone deacetylases with broad-spectrum antitumoral activity against solid and haematological malignancies

**DOI:** 10.1038/sj.bjc.6604025

**Published:** 2007-11-13

**Authors:** J Arts, P Angibaud, A Mariën, W Floren, B Janssens, P King, J van Dun, L Janssen, T Geerts, R W Tuman, D L Johnson, L Andries, M Jung, M Janicot, K van Emelen

**Affiliations:** 1 Oncology Research, Johnson & Johnson Pharmaceutical Research & Development, Turnhoutseweg 30, 2340 Beerse, Belgium; 2 Oncology Research, Johnson & Johnson Pharmaceutical Research & Development, Spring House, PA 19477, USA; 3 HistoGenex, Drie Eikenstraat 661, B-2650 Edegem, Belgium; 4 Institute of Pharmaceutical Sciences, Albert-Ludwigs-Universität Freiburg, Albertstr. 25, 79104 Freiburg, Germany

**Keywords:** HDAC, HDAC inhibitor, R306465, JNJ-16241199, anticancer agent, small molecule

## Abstract

R306465 is a novel hydroxamate-based histone deacetylase (HDAC) inhibitor with broad-spectrum antitumour activity against solid and haematological malignancies in preclinical models. R306465 was found to be a potent inhibitor of HDAC1 and -8 (class I) *in vitro.* It rapidly induced histone 3 (H3) acetylation and strongly upregulated expression of p21^*waf1,cip1*^, a downstream component of HDAC1 signalling, in A2780 ovarian carcinoma cells. R306465 showed class I HDAC isotype selectivity as evidenced by poor inhibition of HDAC6 (class IIb) confirmed by the absence of downregulation of Hsp90 chaperone c-raf protein expression and tubulin acetylation. This distinguished it from other HDAC inhibitors currently in clinical development that were either more potent towards HDAC6 (e.g. vorinostat) or had a broader HDAC inhibition spectrum (e.g. panobinostat). R306465 potently inhibited cell proliferation of all main solid tumour indications, including ovarian, lung, colon, breast and prostate cancer cell lines, with IC_50_ values ranging from 30 to 300 nM. Haematological cell lines, including acute lymphoblastic leukaemia, acute myeloid leukaemia, chronic lymphoblastic leukaemia, chronic myeloid leukaemia, lymphoma and myeloma, were potently inhibited at a similar concentration range. R306465 induced apoptosis and inhibited angiogenesis in cell-based assays and had potent oral *in vivo* antitumoral activity in xenograft models. Once-daily oral administration of R306465 at well-tolerated doses inhibited the growth of A2780 ovarian, H460 lung and HCT116 colon carcinomas in immunodeficient mice. The high activity of R306465 in cell-based assays and *in vivo* after oral administration makes R306465 a promising novel antitumoral agent with potential applicability in a broad spectrum of human malignancies.

Histone deacetylase (HDAC) inhibitors induce cell-cycle arrest, terminal differentiation and apoptosis in a broad spectrum of human tumour cell lines *in vitro*, and have antiangiogenic and antitumor activity in human xenograft models (Kim *et al*, 2001; Johnstone, 2002; Arts *et al*, 2003; Dokmanovic and Marks, 2005; Drummond *et al*, 2005). Several HDAC inhibitors are in clinical development where activity has been observed mainly in haematological malignancies. This has led to the recent approval of vorinostat (SAHA) for the treatment of cutaneous T-cell lymphoma (Duvic *et al*, 2007).

Histone deacetylase inhibitors act on three distinct subclasses of HDAC enzymes: class I, comprising HDAC1–3 and HDAC8, class IIa, comprising HDAC4, -5, -7, -9 and class IIb, comprising HDAC6 and -10. Inhibition of class I HDACs results in the acetylation of nuclear histone proteins, which affects tertiary chromatin structure and leads to the altered expression of genes involved in cell proliferation, apoptosis and differentiation. Class I HDAC activity is key for uncontrolled proliferation of cancer cells, since downregulation of HDAC1 and HDAC3 expression results in increased histone acetylation and inhibition of HeLa cell proliferation (Glaser *et al*, 2003). Similarly, knock-out of HDAC1 causes embryonic lethality in mice and severely impaired proliferation in mouse embryonic stem cells, characterised by an increase in histone 3 (H3) and histone 4 acetylation and expression of the cyclin-dependent kinase inhibitors p21^*waf1,cip1*^ and p27. Upregulation of other class I HDACs (HDAC2 and -3) failed to compensate for the loss of HDAC1, highlighting its unique function in regulating cell proliferation (Lagger *et al*, 2002). Histone deacetylase 8 is also key to tumour cell growth *in vitro*, but instead of impacting on histone acetylation, HDAC8 associates with cytoskeleton proteins and may play a role in smooth muscle contractility. Knockdown of HDAC8 by RNA interference inhibits growth of human lung, colon and cervical cancer cell lines, highlighting the importance of this HDAC subtype for tumour cell proliferation. Histone deacetylase 8 also associates with the inv(16) fusion protein, one of the most frequent chromosomal translocations found in acute myeloid leukaemia (AML), occurring in over 8% of AML cases (Durst *et al*, 2003, Waltregny *et al*, 2005). Recently, HDAC8 was also found to regulate telomerase activity (Vannini *et al*, 2004; Lee *et al*, 2006). In contrast to the class I HDAC family members, class IIa and IIb HDACs are not directly involved in processes that control proliferation and apoptosis in tumour cells. Downregulation of class IIa members HDAC4 and HDAC7 in HeLa cells using siRNA technology did not result in decreased proliferation (Glaser *et al*, 2003). In addition, class IIa enzymatic activity is not intrinsic, but derived from its association with HDAC3 (Fischle *et al*, 2002). Inhibition of the class IIb enzyme HDAC6, which is a tubulin and Hsp90 deacetylase, caused *α*-tubulin hyperacetylation and decreased cell motility, but did not affect cell-cycle progression (Haggarty *et al*, 2003; Zhang *et al*, 2003). Although inhibition of class II HDAC enzymes does not result in antiproliferative activity *in vitro*, it may potentiate antitumoral effects of other anticancer agents such as radiation (through HDAC4) (Kao *et al*, 2003) or proteasome inhibitors (through HDAC6) (Bali *et al*, 2005). The distinct biological roles of HDAC family members strongly suggest that the selectivity profile of HDAC inhibitors will have major consequences on their clinical activities. Evidently, to obtain single-agent antitumoral efficacy with an HDAC inhibitor, potency towards class I HDAC family members will be essential.

In this paper, we describe the identification of a novel hydroxamate-based HDAC inhibitor with high selectivity towards class I HDACs. R306465 showed potent inhibition of HDAC1 and HDAC8 *in vitro* and specifically induced histone acetylation in tumour cells. R306465 did not alter acetylation of the HDAC6 substrate tubulin significantly, in contrast to other HDAC inhibitors in clinical development that are more potent towards HDAC6 (e.g. vorinostat) or act as broad-spectrum HDAC inhibitors (e.g. panobinostat). R306465 demonstrates potent antiproliferative activity against both solid and haematological tumour cell lines. Finally, R306465 inhibits angiogenesis and shows potent antitumour activity after oral administration in human ovarian, lung and colon tumour xenograft models. The compound is currently being dosed in cancer patients, to evaluate its potential applicability in a broad spectrum of human malignancies.

## MATERIALS AND METHODS

### Compounds

R306465, vorninostat, panobinostat and MS-275 were synthesised in-house. The different batches of R306465 used in these studies consistently exceeded a purity of 97% as measured by NMR, LC-MS and elemental analysis. Trichostatin A was purchased from Calbiochem, Merck, Darmstadt, Germany. All compounds were dissolved in dimethylsulphoxide (DMSO) as 5 mM stock solutions and kept at room temperature.

### Cell culture

All cell lines were cultured at 37°C in a humidified incubator with 5% CO_2_. Media and supplements were obtained from Life Technologies, (Merelbeke, Belgium). All cell lines were obtained from ATCC (American Type Culture Collection, Manassas, VA, USA) unless stated otherwise and cultured according to instructions. The human K562A7 leukaemia cell line was a kind gift by Dr H Heyligen (Dr Willems Instituut, Diepenbeek, Belgium) and was kept as a suspension culture in RPMI-1640 medium supplemented with 2 mM L-glutamine, 50 *μ*g ml^−1^ gentamicin and 5% FCS. Primary human HMEC mammary epithelial cells (Clonetics, North Brunswick, New Jersey, USA) were obtained from BioWhittaker (Verviers, Belgium) and cultured in the mammary epithelial cell growth medium supplemented with growth factors and antibiotics as described by the supplier. Human umbilical cord endothelial cells (HUVECs) (Cascade Biologics C-015-10C, Invitrogen, Merelbeke, Belgium) were grown in M-200 media supplemented with Low Serum Growth Supplement (Cascade Biologics M-200-500 and S-003-10, Invitrogen, Merelbeke, Belgium).

### Cell-proliferation assays and combination studies

The effect of HDAC inhibitors on cell proliferation was measured using an MTT (3-(4,5-dimethyl-2-thiazolyl)-2,5-diphenyl-2H-tetrazolium bromide)-based assay (Serva, Heidelberg, Germany). Cells were seeded in Falcon or NUNC 96-well culture plates (Life Technologies, Merelbeke, Belgium) in 100 *μ*l culture medium and allowed to adhere to plastic for 18–48 h. Test compounds were dissolved in DMSO and further dilutions were made in culture medium, with final concentrations of DMSO never exceeding 0.1% (v v^−1^), and incubated for 4 days. Proliferation of NSCLC was assessed using Alamar Blue-based assay. Cells were seeded in COSTAR 384-well culture plates in 45 *μ*l culture medium and allowed to adhere to plastic for 24 h. Alamar blue was added after 72 h, with the exception of HCC-15, HCC-44, NCI-H2122 and NCI-H460, where Alamar blue was added after 90 h and OD values were measured at 4 days following compound exposure. Data are presented as mean±s.d. of three independent experiments. The IC_50_ values were calculated by non-linear regression analysis using SigmaPlot 4.01 software. A BrdU incorporation assay was used to measure HUVEC proliferation. Human umbilical cord endothelial cells were seeded at 10^4^ cells per well in a 96-well plate in F12K 0.2% FCS (Gibco/BRL 21127-022, Invitrogen, Merelbeke, Belgium) and allowed to adhere for 20 h. Subsequently, proliferation was induced by adding low-serum growth supplement (Cascade Biologics Cat no. S-003-10) and compounds were added at the indicated concentrations. The next day, BrdU incorporation was evaluated over a 24-h period using the BrdU kit from Roche according to the manufacturers description (Roche 1 647 229, Vilvoorde, Belgium). For proliferation of haematological cell lines and the combination studies of R306465 and Bortezomib (PS-341, Velcade), tumour cells were plated in 96-well flat-bottom microtitration plates and incubated at 37°C for 24 h before treatment in 180 *μ*l of drug-free complete cell culture medium. Typically, five concentrations (in 1/3 dilution steps; so as not to exceed 30–40% inhibition of cell proliferation as single agent) of R306465 were combined with five concentrations (in 1/3 dilution steps; so as not to exceed 30–40% inhibition of cell proliferation as single agent) of Bortezomib (PS-341, Velcade®). Control cells were treated with the combined respective vehicle. Cells were incubated for 72 h in the presence of combined test substances at 37°C under 5% CO_2_. At the end of treatments, the cytotoxic activity was evaluated by an MTS assay. At least three independent experiments were performed, each result being issued from quadruplicate determination. Dilutions of each test substance as well as distribution to plates containing cells were performed using a Sciclone ALH 3000 liquid handling system. The compound interactions were calculated by multiple drug effect analysis and were performed by the median equation principle according to the methodology described by Chou and Talalayb (1984). The combination index (CI) was calculated by the Chou *et al* equation (Chou and Talalayb, 1984; Chou *et al*, 1994), which takes into account both the potency and the shape of the dose–effect curve. Each CI was calculated with CalcuSyn software (Biosoft, UK) from the mean affected fraction at each drug ratio concentration. For each independent experiment, the median CI was calculated from all relevant CI values corresponding to each drug combination tested.

### Cell-cycle analysis

Human A2780 ovarian carcinoma cells were seeded at 2 × 10^6^ cells per 75-cm^2^ flask and, after 24 h, incubated with the indicated concentration of test compound. To analyse cell-cycle distribution, sub-confluent cells were collected, centrifuged and washed with PBS. The cell pellets were re-suspended in citrate buffer and nuclei were stained with propidium iodide (Sigma-Aldrich, Bornem, Belgium) as described previously (Vindelov *et al*, 1983). Analysis was carried out using a BD-LSR flow cytometer (BD Biosciences, Erembodegem, Belgium) equipped with an argon laser (488 nm excitation). Cell-cycle profiles were analysed using WinList 3D version 4.0 and areas under the curve were calculated using ModFit LT version 3.1 (Verity Software House Inc.).

### Annexin V staining

Human A2780 ovarian carcinoma cells were seeded at 2 × 10^6^ cells per 75-cm^2^ flask and, after 24 h, incubated with the indicated concentration of test compound for 48 h. The cells were subsequently collected, centrifuged and washed with PBS. The cell pellets were stained with Annexin-V-FITC antibody and propidium iodide with a kit from PharMingen, BD Biosciences, Erembodegem, Belgium (cat 66121E).

### Rat aortic ring assay

Angiogenesis inhibitory activity was measured using the *in vitro* rat aortic ring assay (Nicosia and Ottinetti, 1990). Briefly, thoracic aorta were freshly isolated from 1- to 2-month-old Sprague–Dawley rats and 1-mm-long sections (aortic rings) were embedded in fibrin gel clots in six-well plates. Serum-free MCDB-131 media were added to the wells and the cultures were incubated at 37°C in 5% CO_2_. Test compounds were added at the indicated concentrations at day 0 and again together with fresh media on day 2 and day 5 of culture. The ability of experimental compounds to inhibit microvessel outgrowth was compared directly with vehicle-treated control rings. Quantification of microvessel growth following 8 days in culture was performed using an automated image analysis system consisting of a light microscope equipped with a CCD camera and an automated, custom-designed image analysis program (Nissanov *et al*, 1995). Microvessel area, expressed as pixels^2^, was determined for each aortic ring and the per cent inhibition of microvessel area compared to control rings was calculated using the mean values for each treatment group. Statistical differences between experimental groups (five independent rings) were analysed by ANOVA and Dunnett's *t*-test, with a *P*-value of <0.05 or less considered statistically significant.

### Histone deacetylase activity assays

For assessing HDAC1 activity, A2780 ovarian tumour cells were trypsinised and resuspended in RIPA buffer (50 mM Tris–HCl, pH 7.4, 1% NP40, 0.25% sodium deoxycholate, 150 mM NaCl, 1 mM EDTA, 1 mM Na_3_VO_4_ and EDTA-free protease inhibitor cocktail) and sonicated for 20 s. After centrifugation, the cleared extract was used for immunoprecipitation of HDAC1. The protein extract (100 *μ*g) was precleared for 1 h at 4°C with normal mouse IgG serum (Santa Cruz sc-2025, Heidelberg, Germany). Subsequently, the lysate was rotated at 4°C for 4 h with anti-HDAC1 protein A agarose beads (Upstate 06720, Blognost, Heule, Belgium). Histone deacetylase 1 activity was measured by incubating the HeLa nuclear extracts (3 *μ*g, Biomol, Exeter, UK) or immunoprecipitated HDAC1 complexes with an [^3^H]acetyl-labelled fragment of histone H4 peptide (∼50 000 c.p.m.) [biotin-(6-aminohexanoic)Gly-Ala-(acetyl[^3^H])Lys-Arg-His-Arg-Lys-Val-NH_2_] (Amersham Pharmacia Biotech, Piscataway, NJ, USA) in a total volume of 50 *μ*l enzyme assay buffer (25 mM HEPES (pH 7.4), 1 M sucrose, 0.1 mg ml^−1^ BSA and 0.01% (v v^−1^) Triton X-100). Incubation was performed for 45 min at 37°C (immunoprecipitates) or 30 min at room temperature (HeLa nuclear extract). Before addition of substrate, HDAC inhibitors were added at increasing concentrations and preincubated for 10 min at room temperature. After incubation, the reaction was quenched with 35 *μ*l stop buffer (1 M HCl and 0.4 M acetic acid). Released [^3^H]acetic acid was extracted with 800 *μ*l ethyl acetate and quantified by scintillation counting. Equal amounts of HDAC1 were immunoprecipitated as indicated by western blot analysis. Histone deacetylase 1 activity results are presented as mean±s.d. of three independent experiments on a single lysate. For inhibition of human recombinant HDAC8, the HDAC8 Colorimetric/Fluorimetric Activity Assay/Drug Discovery Kit (cat no. AK-508; Biomol) was used. Finally, we measured the activities of different HDAC isotypes present in partially purified extract from rat liver using the fluorescent HDAC substrates MAL (unselective), B61 (selective for HDAC1, and to some extent also converted by HDAC3) and B12 (selective for HDAC6). Assays were performed in duplicate and the s.e. of the IC_50_ was calculated using Graphpad Prism (Graphpad Software) (Heltweg *et al*, 2004).

### Western blot analysis

Human A2780 ovarian carcinoma cells were incubated with the indicated concentrations of HDAC inhibitors. Total cell lysates were prepared and analysed by SDS–PAGE. Levels of acetylated H3 and H4 histones, total H3 protein and p21^*waf1,cip1*^ protein were detected using rabbit polyclonal and mouse monoclonal antibodies, followed by ECL detection (Upstate Biotechnology 06-599 and 06-866, Abcam ab1791, Cambridge, UK and Transduction Laboratories C24420, BD Biosciences, Erembodegem, Belgium). Levels of total and acetylated tubulin were detected using clone DM1A (Sigma T9026) and clone 6-11B-1 (Sigma T6793). Antibodies for Hsp70 and c-raf were obtained from Transduction (610152) and Stressgen, Michigan, USA (SPA-810), respectively. To control for equal loading, blots were stripped and re-probed with mouse monoclonal antiactin IgM (Ab-1, Oncogene Research products, Merck, Darmstadt, Germany). As secondary antibodies, HRP-labelled anti-mouse (sc-2005, Santa Cruz Biotechnology) and anti-rabbit (65-6120, Zymed, Invitrogen, Merelbeke, Belgium) and fluorochrome-labelled anti-mouse (610-131-121, Rockland, Gilbertsville, USA) and anti-rabbit (A21076, Molecular Probes, Invitrogen, Merelbeke, Belgium) were used. Protein–antibody complexes were then visualised by chemiluminescence (Pierce Chemical Co., Perbioscience, Erembodegem, Belgium) or fluorescence (Odyssey) according to manufacturer's instructions.

### p21^*waf1,cip1*^ Promoter activity *in vivo*

To generate an HDAC inhibitor-responsive p21^*waf1,cip1*^ promoter construct, the –1300 to +88 region of the p21^*waf1,cip1*^ promoter was cloned into pGL3-basic-ZsGreen and stably transfected into A2780 ovarian carcinoma cells (Belien *et al*, 2006). For *in vivo* analysis of p21^*waf1,cip1*^ promoter activity, A2780-p21^*waf1,cip1*^ZsGreen cells were injected s.c. (10^7^ cells per 200 *μ*l) into the inguinal region of Nude mice and caliper-measurable tumours were obtained after 12 days. From day 12 onwards, mice were treated orally (p.o.) once with solvent or the indicated dose of R306465 (10 animals per group). Tumours were collected using transcardial perfusion fixation with 4% paraformaldehyde. Cryosections of 10-*μ*m thickness were mounted on glass slides and air-dried for 30 min at room temperature. Bodipy 558/568-phalloidin staining was performed on whole mounts to visualise actin (background cell staining). ZsGreen fluorescence was evaluated with an Axioplan 2 (Zeiss, Germany) equipped with Epiplan-Neofluar objectives and an Axiocam camera. CD-31 (CY3) immunofluorescent labelling was performed on whole mounts to visualise the endothelium of blood vessels. Mounted samples were observed with the LSM510 laser scanning microscope.

### Immunohistochemistry

For immunofluorescent staining of AcH3, cryosections of A2780 ovarian carcinoma were fixed for 2 min in 4% neutral buffered formalin and subsequently for 10 min in ethanol (100%). After blocking with goat immunoglobulin (Jackson Immunoresearch Laboratories, Suffolk, UK) for 60 min, the sections were incubated with anti-AcH3 (Upstate, 1/5000) for 2 h and with goat anti-rabbit CY3-conjugated secondary antibody (1/500 Jackson Immunoresearch Laboratories) for 2 h. For negative controls, primary antibody was omitted. Nuclei were counterstained using Hoechst (1/2000) for 5 min and the cryosections were mounted in glycerin-gelatin. Images were taken using the Zeiss Axioplan 2 & Axiocam, and processed using Photoshop 7.0 and Image Tool Processing Kit. Representative images of each group (*n*=3) are shown. For TUNEL staining, cells were fixed with 4% paraformaldehyde for 5 min and then incubated for 1 h at 37°C in a solution containing 25 mM Tris, 200 mM sodium cacodylate, 1.25 mg ml^−1^ BSA, 1.25 mM CoCl_2_, 10 mM dATP (Sigma Chemical Company), 2.5 mM fluorescein-dUTP (Amersham Pharmacia Biotech AB) and 50 U ml^−1^ TdT (Roche, Vilvoorde, Belgium). Incorporated Fluorescein-dUTP was detected with a sheep antifluorescein peroxidase-conjugated antiserum (Boehringer Mannheim, 1/600, Roche, Vivoorde, Belgium). The peroxidase conjugate was visualised using amino ethylcarbazol.

### *In vivo* antitumoral studies

R306465 was formulated at 2 mg ml^−1^ in 20% hydroxypropyl-*β*-cyclodextrin (final pH 8.7) as an injectable solution. All mice used in the *in vivo* studies were athymic male NMRI nu/nu mice purchased from Janvier (France) and were treated according to the ethical guidelines prescribed by UKCCCR. A2780 ovarian, HCT116 colon and H460 lung carcinoma cells were injected s.c. (10^7^ cells per 200 *μ*l) into the inguinal region of Nude mice. From day 4 onwards, mice were dosed orally daily during 28 days (QDx28, p.o.) with the indicated dose of R306465 (10 animals per group, 0.5 ml per mouse). Tumour size was determined using caliper measurement and tumour volume was determined by using the formula: TV=(a^2^ × b)/2 (in which *a* represents the width and *b* the length).

## RESULTS

### Identification of R306465 as a potent Histone deacetylase inhibitor

**Figure 1:**
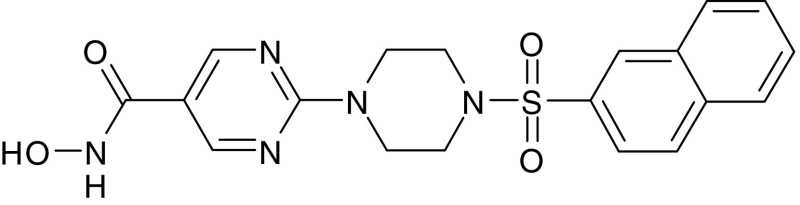
Chemical structure of R306465.

R306465 (Figure 1) was identified as potent novel hydroxamate-based inhibitor of histone deacetylase 1 (HDAC1) inhibiting immunoprecipitated HDAC1 complexes *in vitro* with an IC_50_ value of 3.3 nM and A2780 ovarian tumour cell proliferation with an IC_50_ of 39 nM (Table 1). A comparison of several other HDAC inhibitors currently in clinical development confirmed that panobinostat (LBH589) is also a highly potent inhibitor of both HDAC1 activity and tumour cell proliferation *in vitro* (Bali *et al*, 2005), while vorinostat (SAHA) showed lower potency towards both HDAC1 and tumour cell proliferation, which is in agreement with previous publications (Butler *et al*, 2000). The benzamide MS-275 did not inhibit HDAC1 enzyme activity up to 1 *μ*M, and showed low antiproliferative potency. R306465 inhibited the class I HDAC8 at least 10 times more potently than vorinostat and panobinostat (Table 1). We have not studied cellular activity towards HDAC8 since its natural substrates have not been identified until recently, when Lee *et al* (2006) demonstrated that HDAC8 stabilises ‘human ever-shorter telomeres 1B’ and thereby sustains telomerase activity. Activity towards HDAC1–3 and HDAC8 has been demonstrated to be essential for tumour cell proliferation (Glaser *et al*, 2003).

To study HDAC subtype selectivity of R306465 in further detail, we compared deacetylation of the HDAC1/HDAC3 substrate, B61, and the HDAC6-specific substrate, B12 (Heltweg *et al*, 2004) in a rat liver HDAC preparation. R306465 preferentially inhibited deacetylation of B61, while vorinostat was 9-fold more potent in inhibiting the deacetylation of the HDAC6-specific substrate (Table 1). Panobinostat demonstrated equipotent activity towards both substrates.

Since R306465 differed from other HDAC inhibitors in showing high class I selectivity *in vitro*, we subsequently investigated HDAC subtype selectivity in cells. We evaluated the acetylation status in A2780 ovarian carcinoma cells of H3, which is acetylated through class I HDACs (Glaser *et al*, 2003), and tubulin, which is acetylated by the class II family member HDAC6 (Matsuyama *et al*, 2002; Zhang *et al*, 2003). Inhibition of HDAC6 also induces Hsp90 acetylation, resulting in Hsp70 induction and degradation of Hsp90-associated pro-survival and pro-proliferative client proteins such as c-raf (Bali *et al*, 2005).

**Figure 2:**
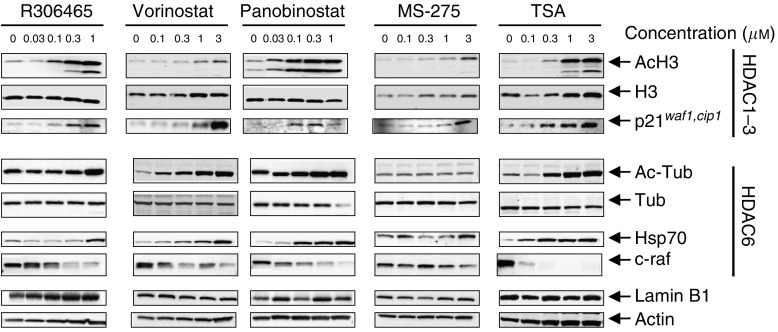
Substrate selectivity of R306465. Human A2780 ovarian carcinoma cells were incubated with the indicated concentrations of R306465, vorinostat, panobinostat, MS-275 or Trichostatin A (TSA) for 24 h. Total cell lysates were prepared and analysed by SDS–PAGE. Levels of acetylated H3 and tubulin, and of total levels of H3 and tubulin and of p21^*waf1, cip1*^ and Hsp70 and c-raf protein, were detected using rabbit polyclonal and mouse monoclonal antibodies, followed by ECL detection as indicated in the Methods section. To control for equal loading, blots were stripped and re-probed with antibodies against actin and the nuclear protein Lamin B1. A representative experiment out of three is shown.

R306465 induced H3 acetylation and p21^*waf1, cip1*^ induction at concentrations as low as 100 nM (Figure 2). Tubulin acetylation and Hsp70 levels, on the other hand, increased only at concentrations as high as 1 *μ*M, indicating a 10-fold higher cellular potency for HDAC1 *vs* HDAC6, reflecting class I selectivity observed *in vitro* (Table 1). Vorinostat on the other hand, induced H3 acetylation only at 1–3 *μ*M, while tubulin acetylation and Hsp70 induction were already evident at 100 nM, indicating a 10-fold higher potency for HDAC6 for this agent. The other hydroxamic acid-based HDAC inhibitors, panobinostat and TSA, inhibited HDAC1/HDAC6 at similar concentrations, since both substrates were acetylated at 100 and 300 nM, respectively. The benzamide MS-275 induced H3 acetylation at 3 *μ*M, but did not affect tubulin acetylation or Hsp70 levels at this concentration. These data confirm that R306465 is a specific inhibitor of HDAC1/3 in A2780 tumour cells, which is in contrast to other HDAC inhibitors in clinical development that are more potent towards HDAC6 (e.g. vorinostat) or have a broader spectrum of activity (e.g. panobinostat).

### R306465 has broad-spectrum antiproliferative activity against solid and haematological cancer cell lines

**Figure 3:**
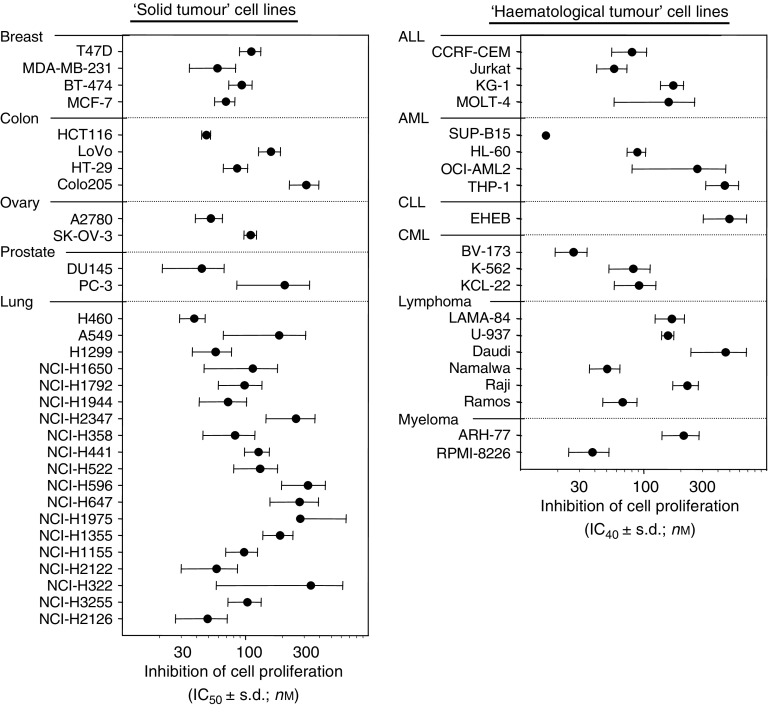
Antiproliferative activity of R306465. Human tumour cell lines were seeded at low cell density and after 24 h cells were incubated with R306465 at 3 × 10^−9^, 10^−8^, 3 × 10^−7^, 10^−6^, 3 × 10^−6^ and 10^−5^ M. The number of viable cells after a 4-day incubation period was assessed using a standard MTT colorimetric assay or Alamar Blue assay as indicated in the Methods section. Values represent mean±s.d. of three independent experiments. For haematological tumour cell lines, IC_40_ values (concentrations leading to 40% inhibition of cell proliferation) were determined for technical reasons: (ALL) acute lymphoblastic leukaemia; (AML) acute myeloid leukaemia; (CLL) chronic lymphoblastic leukaemia – EHEB (chronic B cell leukaemia); and (CML) chronic myeloid leukaemia.

Histone deacetylase 1–3 and HDAC8 have been demonstrated to be key for tumour cell proliferation (Glaser *et al*, 2003). We therefore investigated the antiproliferative effects of R306465 in a broad panel of human tumour cell lines from both solid and haematological origin. As indicated in Figure 3, R306465 inhibited cell proliferation in all lung, breast, colon, prostate and ovarian tumour cell lines tested, with IC_50_ values ranging from 38 to 338 nM. This antiproliferative effect was not dependent on p53 genotypic status, nor ras mutational status in colon and lung tumour cells (data not shown). Similarly, R306465 inhibited proliferation with comparable potency in acute lymphoblastic leukaemia (ALL), AML, chronic lymphoblastic leukaemia (CLL), chronic myeloid leukaemia (CML), lymphoma and myeloma tumour cells (IC_50_ values=15–486 nM). Primary human mammary epithelial cell (HMEC) proliferation was inhibited at similar concentrations as tumour cells (IC_50_=32±9.7 nM) but quiescent, non-proliferative HMEC cells were insensitive to the effects of R306465 (IC_50_=7815±435 nM, data not shown).

Histone deacetylase 6 has been reported to potentiate the effects of proteasome inhibitors (Bali *et al*, 2005). The observation that R306465 mainly affects HDAC1, but not HDAC6 activity, prompted us to investigate whether R306465 could work synergistically with Bortezomib (Velcade). As indicated by the CI in Table 2, R306465 and Bortezomib show potent additivity and synergy in a large panel of haematological tumour cell lines, including ALL, AML, CML, lymphoma and myeloma. These data suggest that activity towards HDAC6 may not be necessary for synergy with Bortezomib.

### R306465 induces apoptosis and inhibits angiogenesis

**Figure 4:**
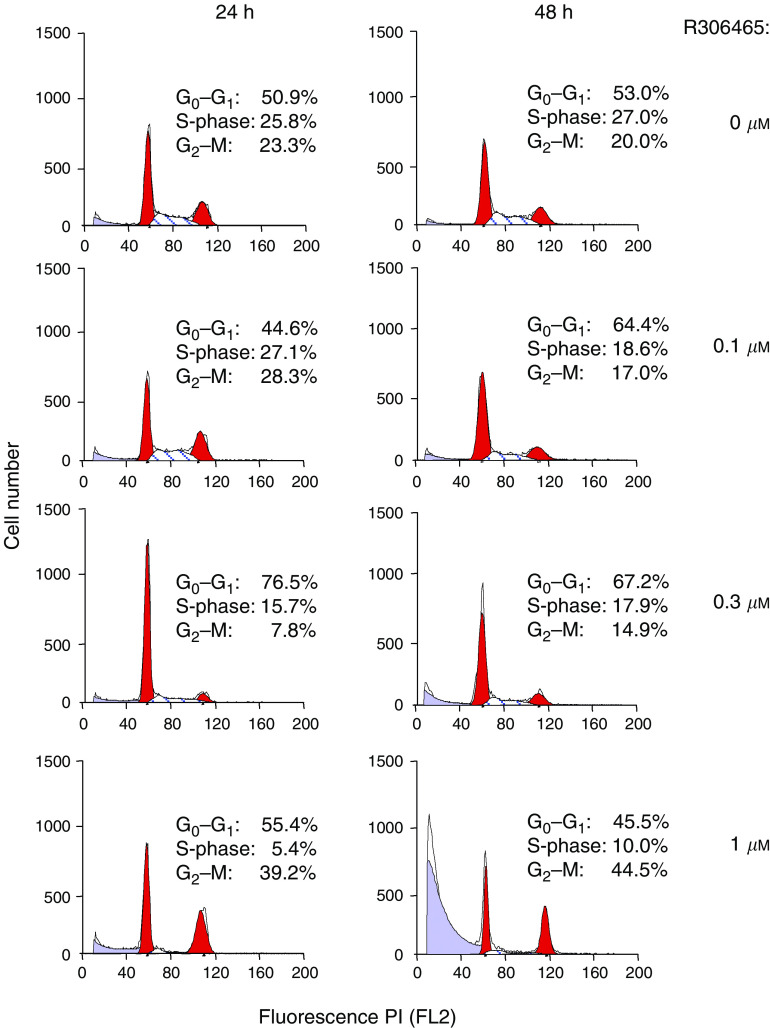
R306465 affects cell-cycle distribution. Human A2780 ovarian carcinoma cells were incubated with the indicated concentrations of R306465 for 24 or 48 h. DNA content of nuclei was evaluated using propidium iodide staining followed by FACS analysis, and the number of cells in sub-G_1_, G_1_, S and G_2_M phase was calculated as a percentage of control. Results are expressed as % of total cells and a representative experiment out of three is shown.

**Figure 5:**
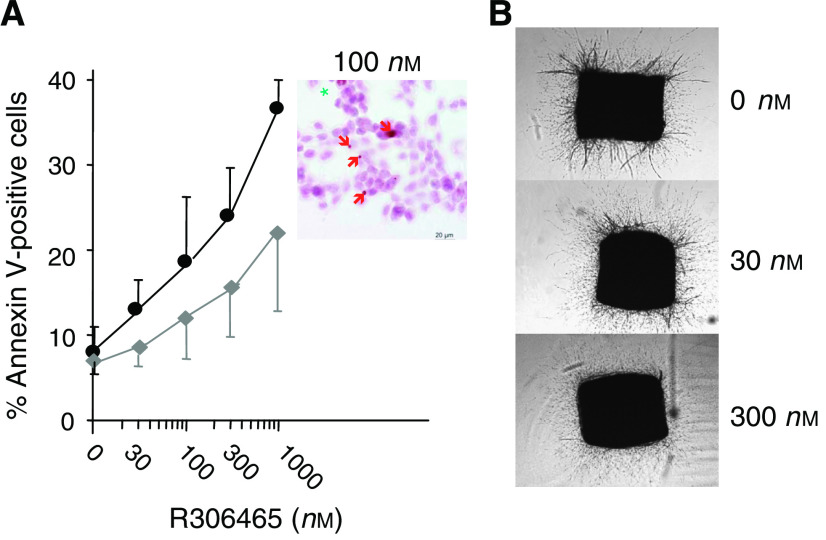
R306465 induces apoptosis in human A2780 ovarian carcinoma cells and inhibits angiogenesis. (**A**) A2780 ovarian carcinoma cells were incubated with the indicated concentrations of R306465 for 48 h. Cells were stained with Annexin V (Pharmingen) and propidium iodide, and analysed by FACS. Apoptotic cells show Annexin V binding on the plasma membrane, but cells are intact and therefore do not stain for propidium iodide. Membrane-damaged cells were defined as both Annexin V- and propidium iodide-positive. Shown are mean values±variation for two independent experiments. Inset: cells incubated with R306465 (100 nM) were stained for DNA breaks using TUNEL dUTP labelling followed by peroxidase staining. Green asterisks represent mitotic cells, while red arrows point at specific DNA fragmentation. (**B**) Rat Aortic Ring Assays were performed as described in the Methods section. Aortic rings were incubated with either solvent or with the indicated concentrations of R306465 for 8 days and the average microvessel area for five independent rings was quantified.

To investigate whether the antiproliferative effect of R306465 in A2780 ovarian tumour cells was due to induction of cell-cycle arrest or cell death, FACS analysis was performed. As shown in Figure 4, after 24 h incubation, a concentration-dependent decrease in S phase (DNA synthesis) was observed at 300 nM, with a parallel increase in G_1_ phase. However, at higher concentrations (1 *μ*M), an increase in the sub-G_1_ fraction of cells was observed. After 48 h, an increase in sub-G_1_ phase was observed at all active concentrations, starting from 100 nM, suggesting that the antiproliferative effect of R306465 is linked to A2780 cell death. R306465 treatment caused a significant increase in the percentage of cells positive for Annexin V in a concentration-dependent manner indicative of apoptosis. An increase in the number of necrotic cells was also observed, which likely represents apoptotic cells in later stages of cell death. Induction of apoptosis was further confirmed by an increase in cells showing DNA fragmentation as assessed by TUNEL staining (inset, Figure 5A). In subsequent experiments, HDAC inhibitors also induced substantial apoptosis in HCT116 and HT-29 colon tumour cells, DU145 prostate cancer cells and H1299 large cell carcinoma, pointing at apoptosis as a major denominator of response *in vitro* (data not shown). R306465 also inhibited angiogenesis as demonstrated by a decrease of microvessel growth in the rat aortic ring assay (Figure 5B). Total microvessel area was significantly inhibited by 67±7% (mean±s.e.m.) at 300 nM compared to controls. The antiangiogenic effect of R306465 is in agreement with its antiproliferative potency for primary human endothelial cells. R306465 inhibited the growth of HUVECs with an IC_50_ of 186±23 nM.

### R306465 induces H3 acetylation and p21^*waf1,cip1*^ promoter activity in A2780 ovarian tumour tissue *in vivo*

**Figure 6:**
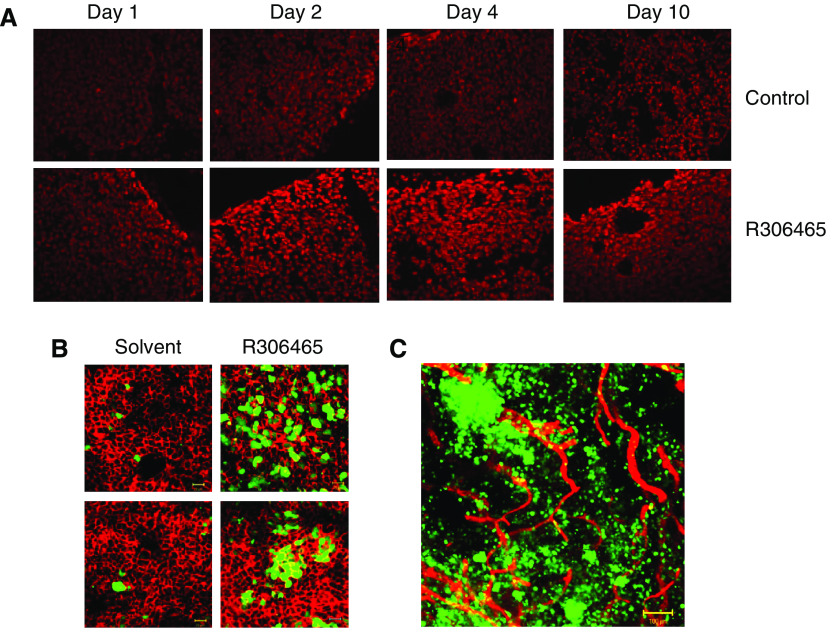
R306465 induces histone acetylation and activates the p21^*waf1,cip1*^ promoter *in vivo.* Nude mice were injected s.c. with A2780 ovarian carcinoma cells and subsequently treated p.o. once daily with vehicle (20% HP-*β*-CD, control) or R306465 at 40 mpk (R306465). (**A**) Tumours were harvested 4 h after the last dose. Nuclei are stained with Hoechst, while (CY3) immunofluorescent labelling was performed to visualise acetylated H3. Representative sections from four independent mice are shown. (**B**) and (**C**) Tumours were collected after 24 h using transcardial perfusion fixation with 4% paraformaldehyde. (**B**) Bodipy 558/568-phalloidin staining was performed on whole mounts to visualise actin (background cell staining). (**C**) CD-31 (CY3) immunofluorescent labelling was performed on whole mounts to visualise the endothelium of blood vessels. Mounted samples were observed with the LSM510 laser scanning microscope.

The antiproliferative and cytotoxic effect of HDAC1 inhibition is linked to increased histone acetylation status. To assess whether R306465 increases H3 acetylation *in vivo*, immunodeficient mice bearing human A2780 ovarian xenografts were treated with R306465 orally once daily at 40 mpk (mg kg^−1^), and tumours were harvested 4 h after dosing at different days. An increase in acetylation was observed at the edge of the tumour tissue 4 h after the first treatment, while on day 2, a maximal and homogeneous increase in H3 acetylation was observed throughout the tumour tissue (Figure 6). To visualise subsequent activation of downstream signalling pathways *in vivo*, we used a fluorescence-based gene expression xenograft model in which green fluorescent protein expression is driven by the promoter of the cyclin-dependent kinase inhibitor p21^*waf1,cip1*^ (Belien *et al*, 2006). Treatment of tumour-bearing mice with HDAC1 inhibitors results in de-repression of the p21^*waf1, cip1*^ gene and induction of fluorescence *in vivo*. As shown in Figure 6A, control tumour sections displayed only a few fluorescent cells. On the other hand, tumour sections treated only once with R306465 at 40 mpk (p.o.), contained a large number of fluorescing cells 24 h after dosing, indicative of p21^*waf1,cip1*^ promoter activation in the ovarian tumour tissue. ZsGreen fluorescence was not uniform throughout the tissue; areas of high fluorescence containing a few to hundreds of cells were surrounded by areas with weaker intensity. The clusters containing cells that responded to the treatment were distributed evenly throughout the peripheral and central parts of the tumour and did not colocalise with tumour vasculature structures (Figure 6A and B).

### R306465 inhibits tumour growth *in vivo* after oral administration

**Figure 7:**
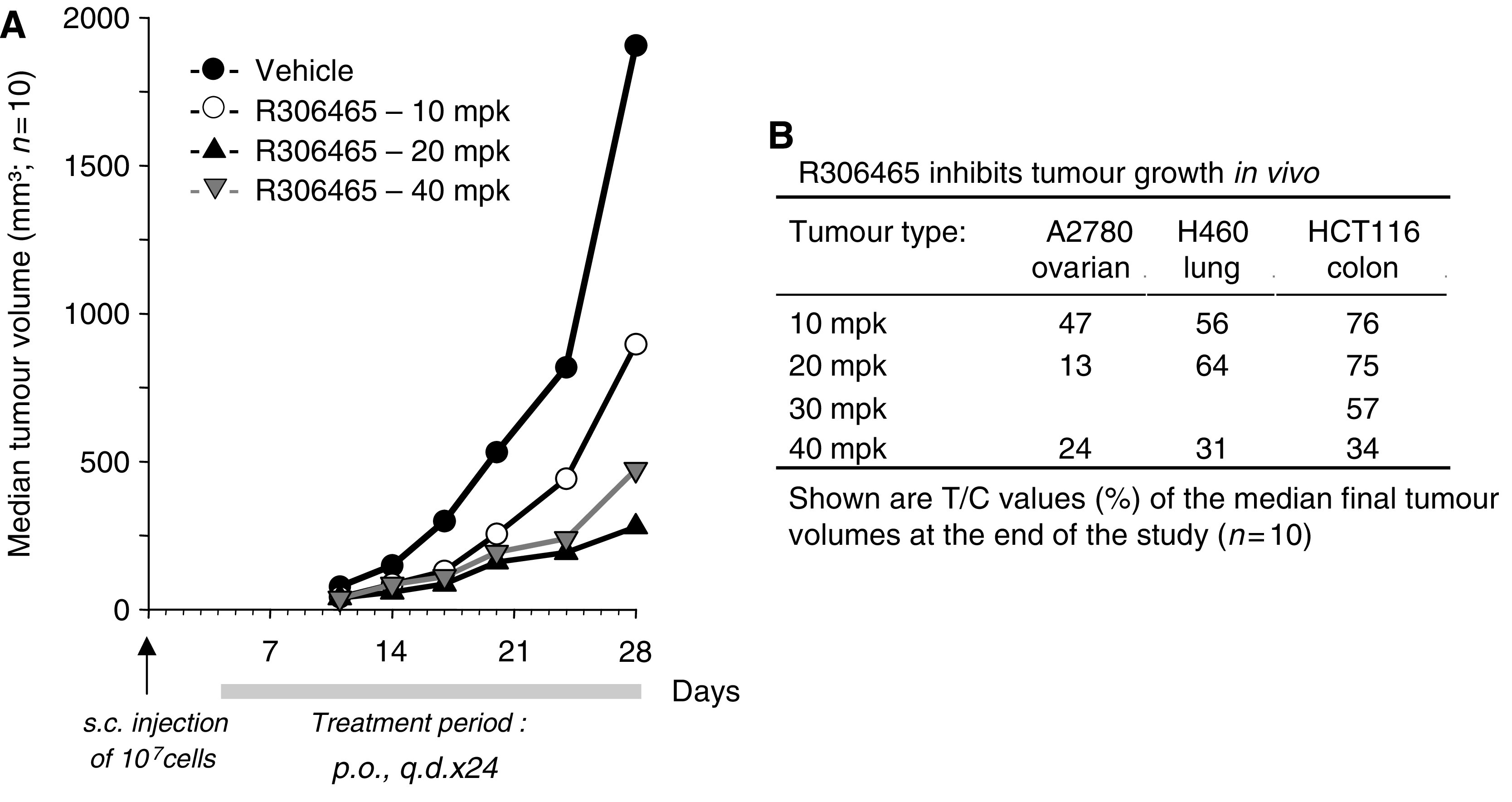
R306465 inhibits tumour growth *in vivo* after oral administration. Nude mice were injected s.c. with A2780 ovarian, H460 lung or HCT116 colon carcinoma cells (10^7^ cells per mouse) and subsequently treated p.o. with vehicle (control group, 20% HP-*β*-CD; •) or R306465 at either 10 (○), 20 (▴) or 40 mpk (▿). Mice were treated orally once daily (q.d.) between day 4 and 28 (A2780) or between day 4 and 32 (H460 and HCT116). (**A**) A2780 ovarian tumours were measured at least twice a week throughout the study, and results are represented as the median tumour size, expressed in mm^3^, of each individual group (*n*=10). (**B**) Mice were treated orally q.d. at the indicated doses. Results are represented as the treated/control values (%) of the median tumour size (*n*=10) at the end of the study.

R306465 administered continuously for 28 days (once daily, up to 40 mpk p.o.) to immunodeficient mice was well-tolerated. As illustrated in Figure 7, a potent time- and dose-dependent inhibition of tumour growth was observed in the A2780 ovarian xenograft model (panel A). Maximal decrease in final tumour volume was found to be 76–87%, which was obtained both at 20 and 40 mpk doses. Potent and dose-dependent antitumoral efficacy for R306465 after oral administration was also observed in lung (H460) and colon (HCT116) xenograft models (Figure 7). The activity of R306465 in a panel of different human tumour xenograft models suggests that this agent may have applicability against a broad spectrum of human malignancies.

## DISCUSSION

In this paper, we describe a novel hydroxamate-based HDAC inhibitor with potent antiproliferative activity in a broad spectrum of cancer cell lines and antitumoral activity after oral administration in ovarian, lung and colon xenograft models. In agreement with the potent inhibition of class I HDAC enzymes, R306465 showed broad-spectrum antiproliferative activity against all main solid tumour indications *in vitro*, including lung, breast, colon, prostate and ovarian cancer cell lines. Similarly, growth of haematological cell lines ALL, AML, CLL, CML, lymphoma and myeloma was potently inhibited. R306465 differs from other hydroxamate-based inhibitors currently in clinical development in that it has preferential activity towards class I HDACs compared to HDAC6. So far, for the HDAC inhibitors in clinical development, HDAC1 selectivity has been reported only for non-hydroxamic acid-based HDAC inhibitors, such as the benzamide MS-275 (Blagosklonny *et al*, 2002; Hu *et al*, 2003; Glaser *et al*, 2004). In our hands, however, MS-275 did not inhibit the activity of HDAC1 complexes precipitated from tumour cells and induced cellular H3 acetylation only at very high concentrations. HDAC1 inhibition by MS-275 *in vitro* has usually been demonstrated using recombinant HDAC1 enzymes (Hu *et al*, 2003). Assessing HDAC inhibitor selectivity *in vitro* must be approached with caution since active enzymes consist of a multiprotein complex containing other HDACs and cofactors. In agreement with our data, Park *et al* (2004) and Kraker *et al* (2003) also observed modest activity of MS-275 while using immunoprecipitated HDAC1 multiprotein complexes from cancer cells. It therefore appears likely that MS-275 inhibits HDAC1 in its endogenously biologically active multiprotein complex less potently than as a recombinant protein. In addition to HDAC1, R306465 also showed high potency i*n vitro* towards HDAC8. Inhibition of HDAC8 is desirable, since knockdown of HDAC8 by RNA interference inhibits growth of human lung, colon and cervical cancer cell lines, highlighting the importance of this HDAC subtype in tumour cell proliferation. Histone deacetylase 8 is also responsible for the transcriptional repression by the inv(16) fusion protein, one of the most frequent chromosomal translocations found in AML, occurring in over 8% of AML cases. Histone deacetylase inhibitors in clinical development were found to be either equipotent against HDAC6 and class I HDACs (panobinostat) or show preferential activity towards HDAC6 (vorinostat). Selectivity of vorinostat towards HADC6 has not been observed previously, possibly because most previous studies were performed with a generic ‘Fluor-de-Lys’ HDAC substrate (Koeller *et al*, 2003; Glaser *et al*, 2004). When using HDAC isotype-specific substrates, R306465 showed poor inhibition of deacetylation of an HDAC6-specific synthetic substrate, which was confirmed while analysing HDAC1–3 (H3) and HDAC6 substrates (Tubulin, Hsp90) in A2780 ovarian carcinoma cells. The modest effect of R306465 on tubulin deacetylation was surprising because this has previously been shown only for hydroxamic acid-based HDAC inhibitors with a very large capping group (Glaser *et al*, 2004), which is not the case for R306465. A unique aspect of HDAC6 is the presence of two catalytic domains: a C-terminal TDAC pocket that possesses *α*-tubulin deacetylase activity (Hubbert *et al*, 2002) and an N-terminal catalytic domain that is likely responsible for Hsp90 deacetylation. A specific inhibitor of the TDAC catalytic domain only partially inhibited total HDAC6 catalytic activity and poorly induced Hsp90 acetylation (Haggarty *et al*, 2003; Bali *et al*, 2005). All HDAC inhibitors examined in this study (R306465, vorinostat, panobinostat and TSA) showed comparable potency towards both catalytic domains of cellular HDAC6, since tubulin acetylation and inhibition of Hsp90 function were observed at similar concentrations in A2780 ovarian carcinoma cells. Since the activity of HDAC6 is not essential for tumour growth, HDAC6 inhibition likely does not contribute to single agent antitumoral activity of HDAC inhibitors. The clinical implication of the preferential activity of R306465 towards HDAC1 compared to HDAC6, however, is difficult to predict. Histone deacetylase 1 is key for tumour cell proliferation, and found to be upregulated in hormone refractory prostate cancer and breast cancer (Kawai *et al*, 2003; Halkidou *et al*, 2004). Although HDAC6 activity does not drive tumour cell proliferation, HDAC6 has been shown to regulate the response to misfolded protein stress and its inhibition has synergistic effects with Bortezomib, which is administered in relapsed multiple myeloma (Kawaguchi *et al*, 2003; Bali *et al*, 2005). We found potent synergy between R306465 and Bortezomib in a broad panel of haematological cell lines indicating that activity towards HDAC6 is not essential for Histone deacetylase inhibitors to work synergistically with Bortezomib. Histone deacetylase 6 also promotes migration and chemotactic movement in response to serum (Hubbert *et al*, 2002; Haggarty *et al*, 2003). These observations have led to the suggestion that HDAC6 may play a role in metastasis and angiogenesis. R306465, however, was found to inhibit angiogenesis potently, presumably through its antiproliferative effects on primary human endothelial cells rather than effects on cell migration.

Analysis of xenograft tumours from R306465-treated mice revealed events consistent with HDAC1 inhibition (Lagger *et al*, 2003). R306465 administration resulted in p21^*waf1, cip1*^ gene activation in both the central and peripheral tumour region, pointing at a rapid distribution of the compound throughout the tumour tissue. Responding cell clusters were distributed evenly over both the peripheral and central parts of the tumour and did not colocalise with tumour vasculature structures. A similar homogeneous increase of H3 acetylation was observed throughout the tumour tissue. Surprisingly, however, nearly all tumour cells showed an increase in nuclear acetylation while the ZsGreen expression appeared in focal spots of high fluorescence surrounded by areas with weaker intensity. The cells showing ZsGreen fluorescence also showed decreased proliferation, as assessed by immunohistological detection of the proliferation marker PCNA, indicating that these cells represent the responding fraction of the tumour (data not shown). These data imply that although increased histone acetylation may be a prerequisite for response, it may not be sufficient to activate downstream signalling pathways *in vivo*. In this regard, it is of interest that H3 acetylation has been commonly observed in HDAC inhibitor-treated patients in the absence of clinical response. Monitoring p21^*waf1, cip1*^ activation rather than the more generic histone acetylation may be a more predictive marker of efficacy.

Summarising, our data show that R306465 not only potently inhibits HDAC1 *in vitro* and in cells, but also affects HDAC1 downstream signalling pathway*s in vivo*. The fact that HDAC1 has been linked to regulation of cell growth and survival does not imply *per se* a rationale to develop subclass-specific inhibitors. However, the distinct biological roles of class I HDACs and HDAC6 strongly suggest that the selectivity profile of HDAC inhibitors may have major consequences on their clinical activity. Evidently, to obtain single-agent antitumoral efficacy with an HDAC inhibitor, potency towards class I HDAC family members will be essential. Histone deacetylase 1 (but not HDAC3, HDAC6, or HDAC8) was recently also demonstrated to be responsible for sensitisation of cells to TRAIL-induced apoptosis (Inoue *et al*, 2006). R306465 represents a new HDAC inhibitor with high selectivity towards class I HDACs capable of potently inhibiting ovarian, lung and colon carcinoma growth in immunodeficient mice after oral administration. These characteristics make R306465 a promising novel oral antitumoral agent with potential applicability in a broad spectrum of human malignancies.

## Figures and Tables

**Table 1:** Inhibition of HDAC isozymes and tumour cell proliferation (IC_50_, nM)

			**Liver extract**			
**Enzyme substrate**	**HDAC1-IP Ac-H4**	**HDAC8**	**MAL_unselective_**	**B61_HDAC1_**	**B12_HDAC6_**	**A2780 proliferation**
R306465	3.31±0.78	23±17	886±85	51±8	1541±290	39±17
Vorinostat	73±26	370±314	132±7	1032±201	150±9	1157±578
Panobinostat	0.23±0.06	283±29	75±4	47±5	89±12	4.6±1.8
MS-275	>1000	>10 000	Inactive	Inactive	Inactive	376±169
TSA	1.67±0.22	135±51	12±1.2	13.3±1.4	20.7±2.6	23±8

Abbreviations: HDAC=histone deacetylase; TSA=trichostatin A

Inhibition of HDAC activity with R306465 (JNJ-16241199), panobinostat, vorinostat and MS-275 was assessed using a number of different assays: for HDAC1 activity assays, HDAC1 was immunoprecipitated from A2780 cell lysates as indicated in the Methods section and incubated with a concentration range of the indicated HDAC inhibitor. Recombinant HDAC8 activity was measured as indicated in the Methods section. Finally, HDAC isotype activity was measured in rat liver extract using an HDAC1-selective (B61), -unselective (MAL) and an HDAC6-selective (B12) substrate (Heltweg *et al*, 2004). A2780 tumour cell proliferation was measured using a standard MTT colorimetric assay as indicated in the Methods section. Results are expressed as average IC_50_ values (nM)±s.d. for three independent experiments.

**Table 2:** Combination of R306465 and Bortezomib in haematological cell lines *in vitro*

**Cell line**	**Origin**	**CI (mean**±**s.d.)**	**Combination analysis**
CCRF-CEM	ALL	0.93±0.11	Additive
Jurkat	ALL	1.32±0.27	Antagonism
KG-1	ALL	0.90±0.20	Additive
MOLT-4	ALL	0.81±0.06	Synergy
SUP-B15	AML	0.75±0.03	Synergy
HL-60	AML	1.01±0.11	Additive
OCI-AML2	AML	0.70±0.17	Synergy
THP-1	AML	0.87±0.06	Synergy
EHEB	CLL	1.07±0.06	Additive
BV-173	CML	0.83±0.06	Synergy
K-562	CML	0.88±0.25	Synergy
KCL-22	CML	0.90±0.20	Additive
LAMA-84	Lymphoma	1.08±0.09	Additive
U-937	Lymphoma	0.65±0.22	Synergy
Daudi	Lymphoma	0.95±0.15	Additive
Namalwa	Lymphoma	0.76±0.05	Synergy
Raji	Lymphoma	0.55±0.02	Synergy
Ramos	Lymphoma	1.31±0.14	Antagonism
ARH-77	Myeloma	0.91±0.09	Additive
RPMI 8226	Myeloma	0.84±0.19	Synergy

Abbreviations: ALL=acute lymphoblastic leukaemia; AML=acute myeloid leukaemia; CLL=chronic lymphoblastic leukaemia; CML=chronic myeloid leukaemia.

Tumour cells were incubated with five concentrations of R306465, combined with five concentrations of Bortezomib (PS-341, Velcade®) for 72 h as described in the Methods section. The combination index (CI) was calculated by the Chou *et al* equation (Chou *et al*, 1994). For each independent experiment, the median CI was calculated from all relevant CI values corresponding to each drug combination tested. Results are expressed as average IC_50_ values (nM)±s.d. for three independent experiments.
